# Notch receptor/ligand diversity: contribution to colorectal cancer stem cell heterogeneity

**DOI:** 10.3389/fcell.2023.1231416

**Published:** 2023-10-04

**Authors:** Morgan Brisset, Patrick Mehlen, Olivier Meurette, Frédéric Hollande

**Affiliations:** ^1^ Department of Clinical Pathology, Victorian Comprehensive Cancer Centre, The University of Melbourne, Melbourne, VIC, Australia; ^2^ Centre for Cancer Research, The University of Melbourne, Melbourne, VIC, Australia; ^3^ Cancer Cell Death Laboratory, Centre de Recherche en Cancérologie de Lyon, INSERM U1052-CNRS UMR5286, Centre Léon Bérard, Université de Lyon, Lyon, France

**Keywords:** colorectal neoplasms, neoplastic stem cells, self-renewal, chemoresistance, posttreatment recurrence, tumor hetereogeneity

## Abstract

Cancer cell heterogeneity is a key contributor to therapeutic failure and post-treatment recurrence. Targeting cell subpopulations responsible for chemoresistance and recurrence seems to be an attractive approach to improve treatment outcome in cancer patients. However, this remains challenging due to the complexity and incomplete characterization of tumor cell subpopulations. The heterogeneity of cells exhibiting stemness-related features, such as self-renewal and chemoresistance, fuels this complexity. Notch signaling is a known regulator of cancer stem cell (CSC) features in colorectal cancer (CRC), though the effects of its heterogenous signaling on CRC cell stemness are only just emerging. In this review, we discuss how Notch ligand-receptor specificity contributes to regulating stemness, self-renewal, chemoresistance and cancer stem cells heterogeneity in CRC.

## 1 Introduction

Cancer is a dynamic disease. During disease progression, tumor heterogeneity generally increases, resulting in the diversification of cell populations within the tumor bulk, each with different phenotypes and distinct sensitivities to chemotherapy (Stanta and Bonin, 2018). This heterogeneity was first thought to emerge solely as a result of expansion and genomic diversification of mutated cells. Indeed, clonal evolution, *i.e.*, genetic and epigenetic changes occurring over time in individual cancer cells, confers a selective advantage to some cells that outgrow others, partly explaining intra-tumoral heterogeneity (Greaves et al., 2012; Stanta and Bonin, 2018). However, this heterogeneity also depends on cancer stem cells (CSCs) present within the tumor. Like normal stem cells differentiating into phenotypically different lineages with limited proliferative potential, CSCs experience epigenetic changes to form phenotypically diverse non-tumorigenic cancer cells that compose the tumor bulk (Shackleton et al., 2009). The cancer stem cell concept was first proposed as an alternative to the clonal evolution of cancer cells to explain intra-tumoral heterogeneity (Nowell, 1976). This model emphasizes the hierarchical organization of cancer cells into phenotypically distinct tumorigenic and nontumorigenic populations. This hierarchical organization results in different subpopulations of cells within the tumor bulk displaying distinct levels or absence of tumor initiating potential. It is now accepted that both clonal evolution and cancer stem cells co-exist to shape intra-tumor heterogeneity (Lyne et al., 2021).

Following chemotherapeutic treatment, sites of recurrence reflect the survival and amplification of drug-refractory cells. CSCs are likely drivers of this process, as intrinsic chemoresistance of CSCs may result in their survival and subsequently lead to their proliferation following treatment cessation (Abdullah and Chow, 2013). Thus, CSC-related heterogeneity fuels chemoresistance of tumor cells and thus recurrence (Zhang A. et al., 2022). Consequently, a precise understanding of the mechanisms underlying this chemoresistance and recurrence via tumor heterogeneity is necessary for the development of effective therapies. Indeed, the contribution of CSCs to intra- and inter-tumor heterogeneity significantly limits the efficacy of cytotoxic and targeted therapies in multiple solid malignancies, such as esophageal, liver, lung, pancreatic, or colorectal cancer (Khatib et al., 2020; Biswas and De, 2021), resulting in low survival rates for patients with advanced disease that cannot be cured via surgical means.

Inter- and intra-tumoral heterogeneity emerge from a combination of tumor cell-intrinsic events and of extrinsic, often microenvironment-driven factors. Intrinsic events include the progressive appearance of new subclones carrying *de novo* mutations (genomic drift) as well as residual epigenetic programming of differentiation pathways, leading to the potential presence of cells varying differentiation states (CSCs, progenitors, specialized differentiated cells) within genetically homogenous subclones. Extrinsic factors include microenvironmental niches and local or systemic regulators of signaling pathways such as the Wnt or Notch pathways (Borovski et al., 2011; Vermeulen et al., 2010; Crea et al., 2009). Along with the regular acquisition of new mutations, these pathways contribute to shaping further heterogeneity within solid tumors, including within CSCs themselves, which can differ according to cell cycle status, surface marker expression, response to chemotherapies, or differentiation potential (Magee et al., 2012; Birbrair, 2019; Tang, 2012). Colorectal cancer (CRC) is a highly heterogeneous disease, providing an ideal background to observe and characterize CSC heterogeneity.

In this review, we will summarize the current knowledge about CSC heterogeneity in colorectal tumors, describe its impact on chemoresistance and post-treatment recurrence, then discuss the relationship between Notch pathway activation, Ligand/Receptor specificity and CSC heterogeneity in colorectal cancer.

## 2 Colorectal cancer

Colorectal cancer is the most commonly diagnosed and the third deadliest cancer worldwide, with almost 2 million new cases and a mortality reaching a million cases each year (Rawla et al., 2019). Main risk factors for this disease include age, family history, inflammatory bowel disease, as well as hereditary syndromes (e.g., Familial Adenomatous Polyposis, Lynch syndrome) (Brenner et al., 2014). Tumor stage, lymph node involvement, and the presence of distant metastases remain the main prognostic factors and guide therapeutic decisions (Labianca et al., 2013).

**FIGURE 1:**
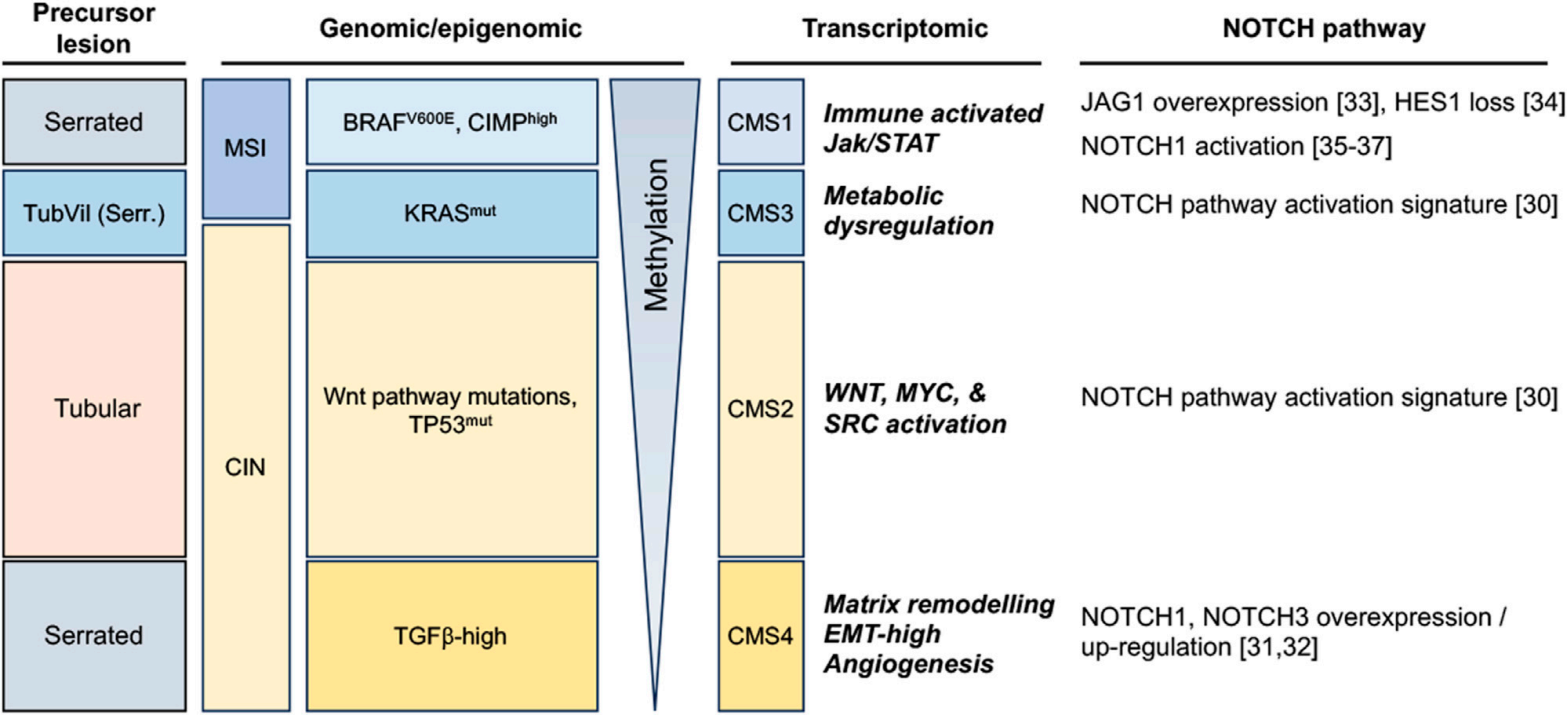
Notch pathway alterations in colorectal tumors, stratified along 4 broad subtypes according to the morphology of precursor lesions and to their most commonly enriched genomic/methylation characteristics and transcriptomic features (TubVil (Serr.): Tubulovillous with serrated features; MSI: Microsatellite Instable; CIN: Chromosomal Instability; CIMP: CpG island methylator phenotype; CMS: Consensus Molecular Subtype).

Colorectal display significant inter-tumoral heterogeneity, underpinned by the existence of different sequences of genomic/epigenomic alterations in different patients, and reflected at the macroscopic level by the emergence of different precursor lesions (summarized in Figure 1). Broadly, preneoplastic colonic lesions (adenomas) can develop along conventional or serrated carcinogenesis pathways. The former manifests itself through the initial development of Tubular, Villous, or Tubulovillous adenomas, while the latter can take either a Traditional or Sessile Serrated appearance, with a small subset displaying pathological features of both (Langner, 2015; Thanki et al., 2017) (Figure 1). Colorectal carcinoma emerging from the conventional carcinogenesis pathway are often driven by activating mutations of genes controlling the Wnt signaling pathway, most frequently through bi-allelic inactivation of the adenomatous polyposis coli (*APC*) tumor suppressor gene, leading to aberrant Wnt pathway activation (Jen et al., 1994; Näthke, 2004). These tumors later develop additional driver mutations, often in genes encoding the KRAS GTPase and the TP53 tumor suppressor, the latter alterations often driving transition from late-stage adenoma to invasive carcinoma (Baker et al., 1990; Naccarati et al., 2012). Conversely, serrated carcinogenesis manifests along two distinct subsets that display significantly different genomic/epigenomic features (Kambara et al., 2004; Yang et al., 2004; Rosenberg et al., 2007). Sessile Serrated lesions emerge from mutations in DNA mismatch repair (MMR) genes that enable microsatellite instability (MSI-High), are enriched for *BRAF* ^V600E^ mutations, and usually exhibit a high CpG island methylator profile (CIMP), resulting in high DNA methylation in resulting tumors. In contrast, traditional serrated adenomas usually display low methylation, a stable microsatellite profile (MSS), may exhibit mutations in KRAS or BRAF, but are mostly characterized by their high level of TGFβ pathway activation (Nguyen et al., 2020; Kim and Bodmer, 2022) (Figure 1).

More recently, a more detailed molecular classification has emerged from the integration of genomic and transcriptomic features of colorectal tumors, resulting in the stratification of this disease into four consensus molecular subtypes (CMS 1–4) (Guinney et al., 2015). Genes and pathways that characterize each CMS have significantly improved our understanding of previously described CRC subtypes and further highlight the inter-tumoral heterogeneity of CRC. Along this classification, CMS1 are characterized by hypermutated DNA, microsatellite instability (MSI-H) and a strong immune activation; CMS2 represents a canonical subtype characterized by chromosomal instability (CIN), by a strongly epithelial signature and an activation of Wnt and Myc pathways; CMS3 is characterized by metabolic dysregulations and an enriched *KRAS* mutation profile; and CMS4 represents a mesenchymal subtype, emerging mostly through the traditional serrated pathway and characterized by stromal invasion and angiogenesis and an activation of the transforming growth factor–β (TGFβ) (Guinney et al., 2015) (Figure 1).

Considering the important role of Wnt signaling in colorectal cancer and the well-documented crosstalk between Wnt and other developmental pathways, the Notch pathway has also been a subject of intense investigation in this cancer. Altered expression or functional activation of Notch receptors and ligands have indeed been reported to play a significant role in colorectal cancer progression, and data is progressively emerging that identifies which CRC subtypes may be more dependent on this pathway (Figure 1). Indeed, it appears that NOTCH1 and NOTCH3 receptors are upregulated in the CMS4 subtype (Jackstadt et al., 2019; Koch and Radtke, 2020). NOTCH3 is associated with tumor staging, lymph node and distant metastasis in this subtype, whereas NOTCH1-signaling correlates with poor prognosis and drives metastasis. Furthermore, both CMS2 and CMS3 subtypes exhibit a Notch pathway activation transcriptomic signature (Guinney et al., 2015). The CRC serrated pathway exhibit Notch pathway dysregulation as well, notably single cell analysis revealed overexpression of the *JAG1* ligand and of *MYC* (Notch target) compared to normal tissue (Zhou et al., 2023). Interestingly, the HES1 effector has been shown to be completely loss between the progression from hyperplastic polyp to Sessile serrated adenoma/polyp (Cui et al., 2016). Besides, it has been shown that the Bone Morphogenetic Protein (BMP) non-canonical activation of NOTCH1 pathway was participating in epithelial-to-mesenchymal transition (EMT) and led to poorer prognosis in mesenchymal-subtype of colorectal cancer (Irshad et al., 2017), a subtype featuring EMT and MSI high signatures (Kim et al., 2021; Singh et al., 2021).

This increased understanding may have future implications on colorectal cancer treatment (Vinson et al., 2016; Tyagi et al., 2020), particularly in the case of treatment refractory and metastatic tumors.

## 3 CRC therapy

The most common treatment for local CRC remains surgical resection, and though this may also be used for some metastatic colorectal cancers (mCRC), chemotherapeutic interventions are more frequent. Different molecules are used as chemotherapies: 5-fluorouracil (5-FU), an anti-metabolite and uracil analogue preventing DNA synthesis and inhibiting cell division (Longley et al., 2003); Oxaliplatin, a platinum-based anti-neoplastic, that exerts a cytotoxic effect by binding to DNA, RNA and some protein structures (Martinez-Balibrea et al., 2015); and Irinotecan a molecule that inhibits cell division by acting on topoisomerase I (Fujita et al., 2015). Patients receive different combinations of these three molecules, as well as Leucovorin, an active metabolite of folic acid and an essential coenzyme for nucleic acid synthesis that enhances the efficacy of 5-FU (Moran, 1989). These combinations are called FOLFOX (a combination of Oxaliplatin, 5-FU and Leucovorin), FOLFIRI (a combination of Irinotecan, 5-FU and Leucovorin) and FOLFOXIRI (a combination of Oxaliplatin, Irinotecan, 5-FU and Leucovorin) (Fakih, 2015). Another class of treatments is under development, namely, targeted therapies. Indeed, several monoclonal antibody-targeted therapies are recommended for mCRC, such as antagonists of the epidermal growth factor receptor (EGFR): cetuximab and panitumumab, or of the vascular endothelial growth factor A (VEGFA): bevacizumab. Those molecules respectively aim at preventing proliferative signaling cascades and at helping to reduce angiogenesis, two important hallmarks of cancer growth (Hanahan and Weinberg, 2011). Unfortunately, most patients with advanced CRC eventually die from to the disease, despite initial response to these therapies, due to post-treatment tumor recurrence (Song et al., 2018). Effective treatment and patient stratification play crucial roles in managing colorectal cancer and improving patient outcomes. Treatment approaches include surgery, chemotherapy, targeted therapy, immunotherapy, and radiation therapy, which are selected based on tumor stage and molecular characteristics (Benson et al., 2021; Van Cutsem et al., 2016). While advancements in treatment have led to improved survival rates, tumor recurrence remains a significant challenge and a leading cause of mortality in colorectal cancer (Tarantino et al., 2015). Recurrence patterns may involve local, regional, or distant metastasis (Weiser, 2018). To address this, personalized treatment strategies are employed, which may include adjuvant chemotherapy, targeted therapies, and close surveillance to facilitate early detection of recurrence (Grothey et al., 2018). Molecular biomarkers, such as *RAS* and *BRAF* gene mutations, guide treatment decisions and help identify patients at higher risk of recurrence (Douillard et al., 2013). Although most adjuvant chemotherapies still appear to have limited effect on recurrence (Kaiser et al., 2006), patient stratification based on molecular profiling and genetic testing enables tailored treatment plans and may provide benefit in the future (Thanki et al., 2017; Sveen et al., 2018). Overall, a multidisciplinary approach, close follow-up, and innovative therapies are essential in mitigating the mortality outcomes associated with tumor recurrence in colorectal cancer (Labianca et al., 2013).

Resistance and recurrence of CRC seems to be linked to specific cell subpopulations. These cells are defined as tumorigenic and able to reproduce tumoral heterogeneity indicating their self-renewal abilities (De Angelis et al., 2019). The characterization of these subpopulations in CRC and the events modulating them is therefore a key objective to enhance overall survival of CRC patients and prevent recurrence after treatment. A crucial factor to understanding CSC dynamics and homeostasis is to decipher the interactions between them and their neighboring environment. Indeed, like normal stem cells, CSCs are influenced by external signals, involving interactions with components of the tumor microenvironment and pathways implicated in cellular development, such as Wnt and Notch (Leedham et al., 2005; Ye et al., 2014). An increasing number of studies indicate that the Notch pathway is involved in modulating stemness in normal and cancer cells (ElShamy and Duhé, 2013; Dejana et al., 2017). The aim of this review is thus to present current knowledge on the impact of Notch signaling on colorectal cancer stem cells and its subsequent implication in resistance to CRC treatment and recurrence.

## 4 Role of CSCS in colorectal cancer heterogeneity

As described previously, CSCs are a major determinant of cancer heterogeneity, representing the source of differentiation processes and phenotypic heterogeneity within genomically homogenous clones. In CRC, CSCs exhibit some of the phenotypic characteristics of non-pathological stem cells and are able to initiate and maintain tumor growth. They play a key role in the metastatic process, in resistance to chemotherapies and in the post-treatment relapse of CRC. This subpopulation of cancer cells is characterized by specific properties such as self-renewal, tumor-inducing and sphere forming abilities, limitless replication due to telomerase activity, and multi-lineage differentiation potential (Prasetyanti and Medema, 2017; Zhou et al., 2017). The expression of some specific stemness markers like CD44, CD133, CD24, EpCAM, LGR5 and the ALDH detoxifying enzyme, or the expression of stemness-related genes such as NANOG, SOX2 or KLF4 can be used to enrich or identify this cell subtype. CSCs may also exhibit an aberrant activation of several developmental pathways such as Notch, Hedgehog and Wnt pathways (Cleophas et al., 2017).

Colorectal CSCs are ideal “seed” cells for metastasis of CRC to distant tissue. Their limitless replication and pluripotency enable them to form tumors that are suitable to new microenvironments, often very different from the primary tumor site. In addition, the heterogeneity originating from the asymmetric division of CSCs facilitates the development of metastases despite new microenvironmental conditions. The complexity of the CSC microenvironment is due to numerous signals and factors promoting or inhibiting the stem cell phenotype. Factors such as hepatocyte growth factor (HGF), prostaglandin E2 (PGE2), bone morphogenetic protein (BMP) positively regulate the maintenance of the CSC pool, whereas other factors will trigger their differentiation (Zeuner et al., 2014).

It is worth noting that different characteristics of CSCs seem to be regulated independently from one another, and events enhancing sphere forming abilities may negatively impact stem cell-related gene expression, or resistance to chemotherapies (Ying et al., 2015). For instance, i) knocking down the expression of genes such as AHNAK2 influences sphere formation without affecting the expression of biomarkers like CD133, while ii) knockdown of genes like HLA-B, CCDC92 and PLIN4 influences CD133 expression without changing self-renewal abilities, and iii) genes like ALK and ALMS1 were reported to influence both traits (Zhang X. et al., 2022). Another example of this phenotypic heterogeneity among CSC-like cells is the expression of the EpCAM (epithelial cellular adhesion molecule) marker. In several studies, we observed a direct link between its expression and stemness-like characteristics, including colony formation, self-renewal, tumorigenicity, invasion, metastasis or chemoresistance (Liu et al., 2014; Xiang et al., 2017; Leng et al., 2018). However, other studies described the opposite, as they found that CRC cells expressing low levels of EpCAM were more motile, invasive, chemoresistant, and highly metastatic (Sacchetti et al., 2021).

In 2022, Liu et al. attempted to decipher the inter-tumoral heterogeneity of cancer stemness by performing an unsupervised clustering based on 26 published stemness signatures. Doing so, they revealed two different clusters of patients (Liu et al., 2022); one with low stemness and the other with high stemness properties. The cluster displaying greater stemness properties possessed a higher proportion of advanced tumors and was characterized by worse overall survival and relapse-free survival. The two clusters exhibited several other genetic and phenotypic differences. Genetically, the stemness-high cluster displayed more copy number deletions, whereas the stemness-low cluster possessed a greater mutational burden. Phenotypically, the stemness-low cluster exhibited a proliferation-related phenotype and abundant immune infiltration as well as predominant mutations in multiple oncogenes and tumor suppressors, such as *TP53*, *SYNE1*, *MUC16*, and transcription enrichment of *PIK3CA* and *GFPT1*, *PTMAP9*, *MOGAT3*, and *DPM3* genes, while the stemness-high cluster was significantly associated with mesenchymal and differentiation features as well as overexpression of *S100A12*, *PGM5*, *FUT6*, *SEMA3C*, and *ADAM33* genes. The difference in stemness level between these clusters could be explained by different proportions of CSCs within these tumors, although they presented distinct genomic alterations in multi-omics analyses, suggesting the existence of various populations of cells capable of self-renewal in CRC, reflecting a level of inter-tumor heterogeneity.

**FIGURE 2:**
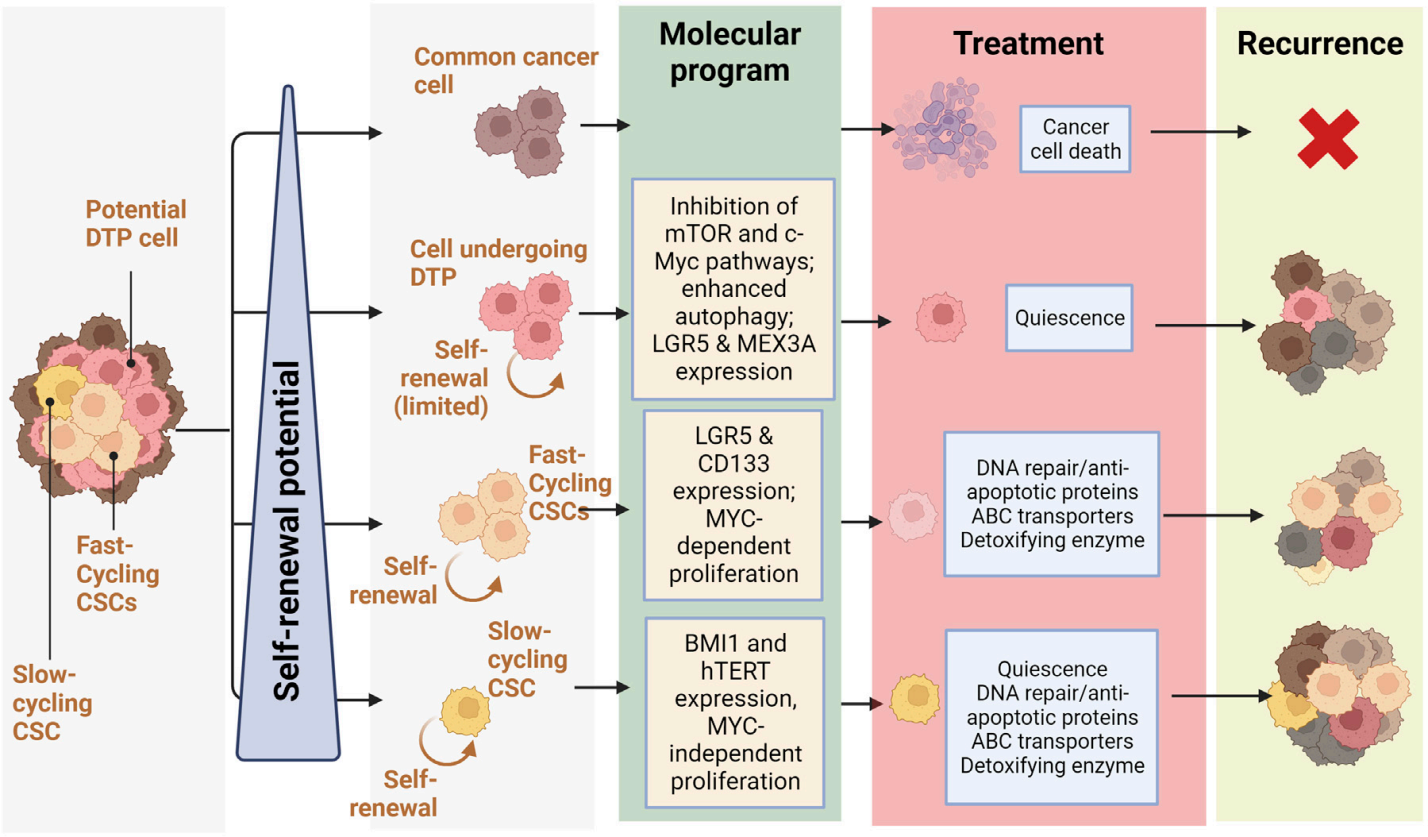
Relationship between self-renewal and recurrence in CRC: Schematic representation of treatment response and recurrence potential by different CRC cells depending on their self-renewal potential (slow-cycling CSCs and fast cycling CSCs and cells undergoing DTP) and the molecular programs they rely on (Quiescence, DNA repair/anti-apoptotic proteins, ABC transporters, Detoxifying enzyme) (Created with BioRender.com).

In 2020 Fumagalli et al. investigated cells at the origin of CRC metastases using a mouse model of CRC and human tumor xenografts. Interestingly, they found that a majority of disseminated CRC cells do not express Lgr5 but form distant metastases in which Lgr5-expressing CSCs progressively arise. This re-expression of Lgr5 by non-CSCs seemed to occur independently of microenvironmental factors and was necessary for metastasis, but dispensable for primary tumor establishment, highlighting the key role of CSC-heterogeneity in important events like metastasis and how the dedifferentiation of non-CSC cells may contribute to this phenomenon (Fumagalli et al., 2020). These findings support the existence of a CSC phenotypic heterogeneity resulting in different populations of stem cells co-existing in colorectal neoplasms. They are also in accordance with the inter-tumoral clusters of stemness observed by (Liu et al., 2022), with high stemness clusters being based on inherent stemness of CSCs and low stemness clusters relying on dedifferentiation of non-CSCs cells. A balance seems to occur in tumors between two different CSC populations: LGR5+ crypt-base columnar stem cells (CBCs) and LGR5-negative regenerative stem cells (RSCs) (Gil Vazquez et al., 2022). CSCs exhibiting markers of CBC, RSC, or both, are capable of self-renewal whereas cells devoid of stem cell markers have very little clonogenic capacity. Despite similar self-renewal capacities, the predominancy of APC and CTNNB1 mutations in CBC-enriched tumors seems to indicate that the CBC stem cell phenotype relies on mutations allowing a ligand-independent activation of Wnt signaling, whereas RSC-enriched tumors display enrichment of KRAS, YAP, TGFβ, and inflammatory pathways (such as IFN-γ). Within neoplasms, an equilibrium between cell populations expressing CBC and RBC markers exists and plastic cells can adaptively shift between these stem cell phenotypes in response to microenvironmental pressures. This heterogeneous and dynamic stem cell population is suspected of being crucial for the adaptive response to selective pressures and to promote lesion outgrowth. Several other studies have demonstrated this apparent heterogeneity of CSC populations. For instance, CD133 expression may not be sufficient to identify the entire CSC population in mCRC, albeit metastatic CD133-expressing cells develop into aggressive tumors and express phenotypic markers of CSCs, notably CD44 (Shmelkov et al., 2008; James et al., 2015). Further evidence of CSC diversity and heterogeneity is the description by Srinivasan et al. of co-existing populations of fast- and slow-cycling colorectal CSCs, undergoing asymmetric division to generate each other (Srinivasan et al., 2016). Fast-cycling CSCs express stemness markers, like LGR5 and CD133, and depend on MYC to proliferate, whereas slow-cycling CSCs are characterized by different markers, such as BMI1 and hTERT, and are independent of MYC (Figure 2). We hypothesize that the inter-tumor heterogeneity characterised by Liu et al. could also result from different proportions of CSC subpopulations within tumors, the stemness-high cluster arising from tumors developed from slow-cycling CSCs and the stemness-low cluster corresponding to tumors originating from fast-cycling CSCs.

Several studies are now trying to combine stemness with other sources of intra-tumoral phenotypic heterogeneity and variability, such as those derived from the tumor microenvironment (Magee et al., 2012; Almendro et al., 2013; Kreso and Dick, 2014). This latter plays a major role in the induction and maintenance of stem-like phenotypes in colorectal tumors. For instance, the specific localization of cells within a tumor was shown to be a key parameter in the pluripotency potential of CRC cells. Indeed, cellular clones responsible for tumor outgrowth in colon cancer predominantly originate from the outer tumor regions (Lenos et al., 2018). Tumor cells from these regions demonstrate higher clonogenic abilities independently of the markers they express. The increased contacts of cancer cells in these regions with components of the microenvironment, such as CAFs, would enhance clonogenic abilities. This is due to the release in the extracellular matrix (ECM) of stemness enhancing proteins such as osteopontin (Lenos et al., 2018) or Netrin-1 (Sung et al., 2019; Brisset et al., 2021). ECM has also been shown to influence stemness through biochemical and biophysical aspects (Nallanthighal et al., 2019). Collectively, these findings demonstrate that functional CSCs differ from cells expressing stemness markers and that the environment prevails over cell-autonomous features in determining stem cell functionality.

This impact of microenvironment further highlights stemness as a phenotypic state fueled not only by cell-intrinsic characteristics but also highly dependent on contextual conditions. Indeed, multiple environment-sensitive epigenetic parameters significantly influence stemness in colorectal CSCs, including DNA methylation, histone acetylation, histone methylation, ubiquitination, and microRNAs (Zeuner et al., 2014; Paschall and Liu, 2015).

Recent studies describe a subpopulation of CRC cells undergoing a phenotypic change following chemotherapy. Indeed, these cells described as “Drug Tolerant Persister” (or DTP) undergo a reprogramming similar to diapause, a reversible state defined by quiescence triggered by unfavorable environmental conditions, such as nutrient deprivation, during embryonic development (Rehman et al., 2021). In contrast to CSCs, all tumor cells have been shown to have an equipotent capacity to become DTPs, which cannot be determined based on genetic mutations but rather on epigenetic changes resulting in a decrease in mTOR pathway activity, alterations in chromatin modifications, hypo-transcription, and an increase in autophagy (Bulut-Karslioglu et al., 2016). Colorectal DTP cells express the genes LGR5 and MEX3A, represent a small subpopulation of the parental tumor (0.3%–5%), are chemoresistant and regenerate after therapeutic treatment (Álvarez-Varela et al., 2022) (Sharma et al., 2010; Liau et al., 2017) (Figure 2). In CRC mouse models, DTP cells displayed a marginal contribution to metastatic outgrowth; nevertheless, after treatment, DTP cells produced cell clones that regenerated the tumor. Conversely, LGR5+ MEX3A-cells were shown to differentiate towards a goblet cell-like phenotype and display no evidence of chemoresistance. It is believed that the DTP subpopulation, unlike CSCs, is not defined by genetical features but distinguished by unique transcriptional and epigenetic profiles, though they share several properties. For instance, DTP cells display fast-cycling and slow-cycling, reminiscent of the balance between CSC populations existing in CRC tumors.

Altogether, these studies put in evidence the plurality of pathways leading to self-renewal, tumor-induction and chemoresistance and explain the variability in expression of stemness markers, or phenotypic characteristics observed among colorectal CSCs. Moreover, the ability of non-CSCs to acquire stem-like characteristics and become CSCs through the process of de-differentiation depending on genetic, epigenetic, or microenvironmental alterations, increase global phenotypic intra-tumoral heterogeneity and complexify the characterization of the different pools of self-renewing cells (Minami, 2015). Thus, dedifferentiation of non-CSCs driven by TGF-beta signaling was shown to enhance stemness and self-renewal abilities in CRC (Nakano et al., 2019). These dedifferentiated non-CSCs seemed able to acquire most phenotypic characteristics of CSCs, including expression of stemness-associated genes, such as LGR5 or CD44, and high tumorigenic potential, following priming by microenvironmental factors shaping CSCs niches (Borovski et al., 2011; Vermeulen et al., 2010) or induction of epithelial–to-mesenchymal transition (EMT) (Morrison and Spradling, 2008). The existence of inter- and intra-tumoral heterogeneity of CSCs in colorectal cancer is now known and represents another challenge for the effective treatment of CRC by chemotherapy. Previously mentioned CSC clusters exhibiting various phenotypic traits as well as differences in response to treatment results in wide range of cellular behavior, impeding prognosis and chemotherapies efficacy. A holistic understanding of the different CSC subpopulations and of signaling pathway alterations responsible for their resistance to chemotherapies is needed to enhance treatment efficacy and prevent tumor recurrence.

## 5 CSCS and chemoresistance

A growing body of evidence is emerging linking CSCs to tumor resistance to treatment and relapse. Indeed, therapies such as radiotherapies or chemotherapies are effective against differentiated and proliferative cancer cells but have an exceptionally low impact on CSCs (He et al., 2014). In colon carcinoma cells, apoptosis triggered upon chemotherapy is comparably lower in CSCs than in differentiated cancer cells (Colak et al., 2014). CSCs resist conventional chemotherapy by escaping DNA damage induced by therapies. Consequently, drugs inducing cell death by targeting DNA, such as cisplatin, oxaliplatin (DNA cross-linkers), methotrexate (DNA synthesis inhibitor), doxorubicin and daunorubicin (topoisomerase inhibitors) are inefficient against CSCs (Peitzsch et al., 2013). CSC resistance to DNA damage is modulated by several mechanisms, including alteration of cell cycle checkpoints (Solier et al., 2012), high expression of DNA damage repair proteins (such as p-ATM) (Khanna et al., 2001; Sánchez-Flores et al., 2015; Anuja et al., 2019), modified metabolism (Blondy et al., 2020), and an efficient scavenging of reactive oxygen species (ROS) induced by chemotherapy or radiotherapy (Diehn et al., 2009).

Increasing data suggest that quiescence contributes to CSCs chemoresistance, as some of the cytotoxic drugs mainly target highly proliferative cells (Is et al., 2015; Das et al., 2019; Das et al., 2020). After treatment, quiescent CSCs can resume their cell cycle and fuel tumor regrowth through the activation of signaling pathways fostering cell growth and proliferation (Francescangeli et al., 2020). The mechanisms underlying colorectal CSC quiescence are not yet fully understood, though the expression of transcription factors such as c-YES (Touil et al., 2014), ZEB-2 (Francescangeli et al., 2020) or HMGA-1 (Puca et al., 2014) were proven to be crucial for the entry of CSCs into the reversible quiescent G0 cell-cycle phase.

CSCs exhibit an aberrant and flexible metabolism driving epigenetic and genetic changes necessary for tumor onset, development, intra-tumoral heterogeneity, and recurrence (Kaur and Bhattacharyya, 2021). The metabolic plasticity of CSCs allows them to switch between glycolysis and oxidative metabolism (OXPHOS) to adapt to microenvironmental stresses like nutrients deprivation and therapies (Kahlert et al., 2017). For instance, CSCs rely less on ATP and glucose-dependent lipid synthesis, suggesting that metabolic plasticity of CSCs could play a key role in their quiescence and consequently in their resistance to cytotoxic agents (Vincent et al., 2014). Moreover, 5-FU being an inhibitor of thymidine synthesis, it was reported to have a limited efficacy against CSCs due to their capacity to switch to high levels of oxidative phosphorylation, characterized by a high expression of pyruvate kinase M1 (PKM1) and repression of PKM2, and resulting in inhibition of the pentose phosphate pathway (Denise et al., 2015; Vellinga et al., 2015).

Aldehyde dehydrogenase (ALDH) is an enzyme catalyzing the oxidation of endogenous and exogenous aldehyde substrates, a key function of cellular detoxification (Tomita et al., 2016). Members of the ALDH1 family, such as ALDHA1, ALDHA3 or ALDHB1, are strongly active in normal tissue stem cells and are considered to be markers of colorectal CSCs (Khorrami et al., 2015; Feng et al., 2018). The ability of ALDH to detoxify the active aldehydes formed by reactive oxygen species (ROS) protects CSCs from chemotherapy by increasing the level of ROS (Vishnubalaji et al., 2018).

ABC (ATP Binding Cassette) transporters is a family of large transmembrane proteins relying on ATP hydrolysis to reject metabolites, foreign bodies, and toxic substances from cells (Amawi et al., 2019). In colorectal CSCs, the common ABC transporters, ABCB1, ABCC1, and ABCG2, are highly expressed and actively excrete anti-tumoral drugs, decrease intracellular concentration of drugs, decrease the effect of chemotherapies, and result in multi drug resistance (MDR) (Kangwan et al., 2016; Butler et al., 2017).

Hence, resistance of colorectal CSCs to commonly used therapies is multi layered and relies on a high expression of DNA repair proteins, an acquired quiescence, an adapted metabolism, and expression of ALDH detoxifying enzyme and ABC transporters. These factors explain the resistance of colorectal CSCs against classical chemotherapy regimens like FOLFOX or FOLFIRI (Ilmer et al., 2016).

Furthermore, it is increasingly acknowledged that DTPs are key drivers of chemoresistance and tumor recurrence (Figure 2). As seen above, DTP cells exhibit gene expression patterns that mimic hormonally- and chemically-induced diapause upon chemotherapy. This diapause-like DTP state is characterized by the inhibition of mTOR and c-Myc pathways, as well as enhanced autophagy (Bulut-Karslioglu et al., 2016). By doing so, DTP cells display resistance to a wide variety of chemotherapeutic agents (De Angelis et al., 2019), radiotherapy (Zhao Y. et al., 2020), and targeted therapies (Cabanos and Hata, 2021). Although these DTP-cells revert to a chemo sensitive state after treatment withdrawal (Recasens and Munoz, 2019), tumors in a DTP state may act as a transient reservoir for the development of genetically resistant clones (Ramirez et al., 2016), demonstrating the importance of non-genetic heterogeneity in chemoresistance and recurrence.

As stated previously, intra-tumoral heterogeneity of CSCs in colorectal cancer has been established and contributes to chemoresistance and recurrence in CRC. The balance between the different subpopulation of CSCs and their direct interconversions undoubtedly allows a more diverse response to chemotherapeutic molecules and has been shown to come back to a steady-state post-treatment. For instance, despite the low frequency of slow-cycling colorectal CSCs, this long-lasting subpopulation might constitute a more chemoresistant reserve population needed for recurrence and tumoral repopulation after treatment (Srinivasan et al., 2016) (Figure 2). NOTCH1 signaling through HES1 and HES5 modulation was shown to be the key regulator of asymmetric division, modulating the balance between these two populations, thus directly impacting heterogeneity-based chemoresistance.

In conclusion, phenotypic heterogeneity of CSCs and of normal cancer cells is a driver of resistance to treatment and recurrence in CRC (Figure 2). This pluripotent cell-related heterogeneity fosters a highly adaptive response to chemotherapeutic agents, driving chemoresistance and the reconstitution of tumoral hierarchy after treatment, and is finely tuned by the modulation of molecular signaling, notably the Notch pathway (Ebrahimi et al., 2023).

## 6 Notch pathway general description

Notch signaling orchestrates several major aspects of tumor development by regulating differentiation, proliferation, and apoptosis.

There are four different Notch receptors (NOTCH1, -2, -3 and -4) in mammals. They undergo several post-translational modifications in the endoplasmic reticulum and Golgi apparatus such as cleavage by furins and glycosylation that are responsible for the different affinities of the Notch receptors for their ligands (Moloney et al., 2000; Vinson et al., 2016). The extracellular domains of Notch receptors contain a specific number of epidermal growth factor (EGF)-like repeats for each receptor: 36 for NOTCH1 and -2, 34 for NOTCH3 and 29 for NOTCH4. These EGF domains are essential to prevent ligand-independent activation of the receptors and allows homodimerization upon ligand interaction (Sakamoto et al., 2005). EGF motifs are followed by the Negative Regulatory Region (NRR), including a cysteine-rich LIN-12/Notch-related region (LNR) and two heterodimerization domains, named HD-N and HD-C (Fortini et al., 1993). Prior to being located at the membrane, NOTCH proteins are cleaved by furin-like convertases at site 1 (S1), converting the NOTCH protein into a NOTCH extracellular domain/transmembrane and intracellular domain heterodimer linked by non-covalent interactions (Ntziachristos et al., 2014). The NOTCH intracellular domain (ICD) is a transcriptional activator consisting of ankyrin repeats, a RPBj-Associated-Module (RAM) domain, a transactivation domain (TAD), a nuclear localization signal (NLS) and a PEST (proline-, glutamate-, serine- and threonine-rich region) domain modulating protein stability and proteolytic degradation. The structural differences of Notch receptors confer distinct functions, for instance, NOTCH1 ICD is a strong activator of the Hes1 promoter, while the NOTCH3 ICD appears to be a weaker activator and can even repress NOTCH1-dependent HES activation in certain contexts (Beatus et al., 1999). Though, these distinct effects due to structural differences remain controversial. Indeed, studies by Liu et al., intending to decipher the impact of structural differences of Notch receptors on downstream signals failed to observe a significant structural-based difference between NOTCH1 and NOTCH2 receptors, as the switch in their ICD had no significant effect on carcinogenesis or on the development of organs in which either NOTCH1 or NOTCH2 have dominant roles (Liu et al., 2015).

Ligands of the NOTCH pathway are transmembrane proteins of the Delta/Serrate/LAG-2 family (DSL). In humans, five members of this family are described, three delta-like ligands DLL1, DLL3, and DLL4, and two jagged proteins JAG1 and JAG2 (Meuret et al., 2020). Interaction between the NOTCH receptor of the signal-receiving cell and ligands at the membrane of neighboring cells triggers the unfolding of the Notch regulatory region and unmasks the cleavage sites for ADAM10/17 and the *γ*-secretase, allowing the release of the NOTCH ICD (NICD) into the signal-receiving cell cytoplasm (Langridge and Struhl, 2017). In the nucleus, NICD binds to the CBF-1/Su(H)/LAG1 (CSL) transcription factor and recruits the transcriptional co-activator mastermind-like (MAML) (Kopan and Ilagan, 2009). The targets of this complex are the HES and HEY genes (also called HESR, CHF, HRT, HERP or gridlock), which encode for transcriptional regulators of the basic helix-loop-helix class which is mainly believed to act as repressors (Fischer and Gessler, 2007). HES and HEY proteins both dimerize to provide an intrinsic transcriptional repression activity. HES activation leads to the recruitment of the corepressor TLE/Groucho, whereas HEY confers marginal repression through the recruitment of the mSin3 complex containing histone deacetylase HDAC1 and the corepressor, N-CoR (Iso et al., 2001; Fischer and Gessler, 2007). The main difference between HES and HEY proteins is the lack of the WRPW tetrapeptide in the HEY proteins, preventing the binding of TLE corepressors.

Actors of Notch signaling, such as Notch receptors (NOTCH1, -2, -3 and −4), Notch ligands (JAG1 and -2, Dll-1, -3 and4), as well as Notch pathway targets (HES and HEY effector proteins) are aberrantly overexpressed in colorectal tumors. More precisely, at least 86% of colorectal cancers and 56% of adenomas display an overexpression of genes in the Notch pathway (Shaik et al., 2020). Moreover, the expression of NOTCH1, -3 and -4 receptors was reported to be significantly higher in colorectal cancers compared to normal and adenoma tissues (Zheng et al., 2015), and patients overexpressing NOTCH3 and NOTCH4 receptors, and the HEY1 transcriptional target, exhibit poorer overall survival (Rallis et al., 2019; Shaik et al., 2020). Finally, a functional crosstalk between Notch signaling the self-renewal regulating pathway Wnt has been well-documented in this cancer (Fre et al., 2009; Pannequin et al., 2009; Rodilla et al., 2009), suggesting a possible role for Notch pathway overactivation during colorectal tumorigenesis and possibly in the regulation of colorectal CSCs.

## 7 Notch pathway and colorectal cancer stem cells

Notch signaling is known to control cell fate decisions and stem-cell phenotypes. Alterations in *NOTCH* genes or in genes that regulate the specific signaling resulting in constitutive activation of the pathways, have been observed in CRC (Reedijk et al., 2008). Activation of Notch pathway was shown to be a key regulator of stemness, pluripotency and self-renewal in a vast majority of solid cancers, as well as in patients with chemoresistance (Artavanis-Tsakonas et al., 1999; Espinoza et al., 2013; Yuan et al., 2015). Moreover, Notch ligand/receptor specificity was reported to influence tumor heterogeneity. For instance, in small cell lung cancer (SCLC), NOTCH1/DLL1 signaling clearly influence the balance between two populations of cells (Lim et al., 2017). Indeed, endogenous activation of Notch signaling regulates the switch between “non-neuroendocrine” SCLC cells displaying a high expression of HES1 effector, of Notch receptors, CD44 marker and of mesenchymal markers as well as sphere forming abilities, and “neuroendocrine” SCLC cells expressing high levels of DLL4 and the epithelial marker EpCam as well as a stochastic expression of Notch receptors. The non-neuroendocrine SCLC cells are quiescent and exhibit an enhanced chemoresistance, suggesting similarities with CSCs (Lim et al., 2017). The role of Notch in intra-tumoral heterogeneity in solid tumors is therefore known. Considering the importance of CSC subpopulation heterogeneity within a tumor in CRC resistance to treatment and post-treatment recurrence, we hypothesize that Notch signaling and the diversity of its pathways play a role into CSC diversity.

Notch signaling is well-known for its implications in stemness of intestinal crypt progenitors. For instance, the deletion of *NOTCH1* and *NOTCH2* or inhibition of global Notch activation by treatment with a *γ*-secretase inhibitor triggers colon columnar stem cells to differentiate into goblet secretory cells (van Es et al., 2005; Riccio et al., 2008). The aberrant activation of Notch receptors in CRC, as well as the well-known implications of these pathways in the induction and maintenance of pluripotency in intestinal crypt progenitors, indicate that activation of Notch signaling is involved in the modulation of stemness in CRC cells (Fre et al., 2005; Demitrack and Samuelson, 2016).

**FIGURE 3:**
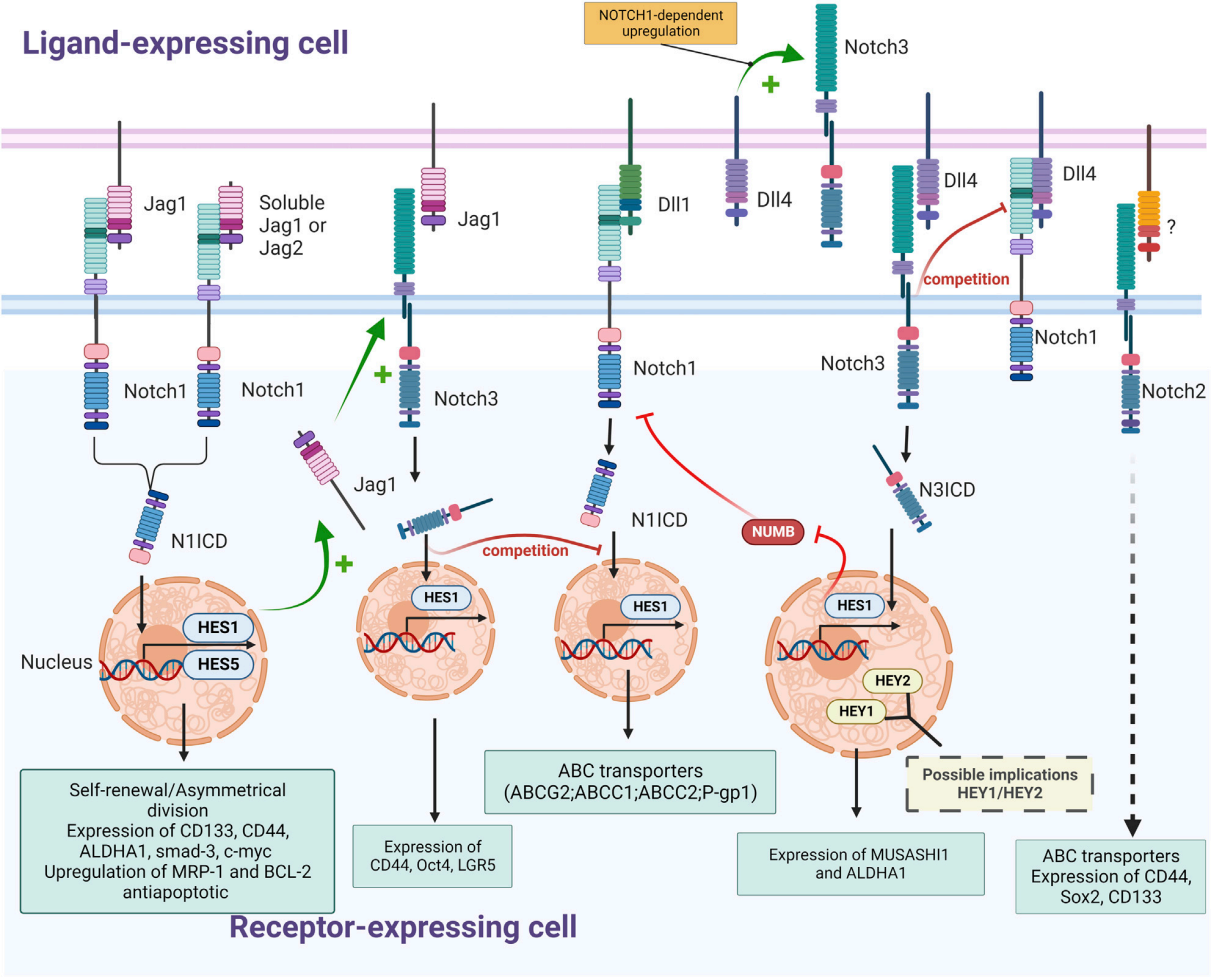
Schematic representation of interactions between NOTCH receptors (NOTCH1, NOTCH2 and NOTCH3) and ligands (Jagged1, DLL1 and Dll4) in CRC and their effects on colorectal CSCs (Self-renewal; expression of stemness marker, ABC transporters and detoxifying enzymes; upregulation of antiapoptotic proteins); The nature of regulatory mechanisms (+: stimulation; -: inhibition; ? and dashed arrow: not described) and of downstream effectors (yellow font: suspected; pale blue font: experimentally validated) is indicated (Created with BioRender.com).

In a pathophysiological context, genes of canonical NOTCH signaling components, such as JAG1, JAG2, and NOTCH1 and the target genes of this pathway, notably HES1, HES4, and HES6, are all significantly higher in CSCs (Sikandar et al., 2010). For instance, JAG1/NOTCH1/HES1 signaling plays an important role in the maintenance and viability of CSCs through the inhibition of apoptosis and cell cycle arrest (Figure 3). Non-specific inhibition of Notch pathway via the use of gamma-secretase inhibitors (GSI) like DAPT ((N-[N-(3,5-Difluorophenacetyl)-L-alanyl]-S-phenylglycine t-butyl ester) activates the intrinsic apoptotic pathway, causing cleavage of caspase-3 and increases levels of proteins responsible for cell cycle arrest, notably ATOH1, p27, and p57 (Sikandar et al., 2010). Notch is also critical for the intrinsic maintenance of colorectal CSC self-renewal and the repression of secretory lineage differentiation-related genes like *MUC2*, demonstrating its involvement in tumorigenicity of colorectal CSCs (Sikandar et al., 2010). Similarly, treatments with ADAM17 inhibitors such as MEDI3622 or TAPI-2, have shown similar negatives effects on self-renewal of colorectal CSCs (Wang et al., 2015; Dosch et al., 2017; Li et al., 2018). Interestingly, a specific inhibition of the delta ligand DLL4 with a neutralizing antibody results in similar defects in colorectal CSCs (Fischer et al., 2011). Indeed, upon treatment with anti-DLL4, colorectal CSCs exhibit reduced self-renewal abilities and higher levels of differentiation promoting the expression of proteins like ATOH1 and CHGA through HES1 upregulation (Table 1).

**TABLE 1:** Effect of Notch pathway on CSCs and CSCs-related chemoresistance: Summary of previous studies addressing specific effects of NOTCH ligands and receptors, as well as NOTCH pathway effectors, on CSCs (stemness marker, anti-apoptotic protein and drug-efflux transporters expression), chemoresistance (resistance to drugs and expression of recurrence) and pluripotency (Self-renewal, tumorigenicity, and differentiation), as well as their interaction with other NOTCH pathway members.

Ligand/Receptor (effector)	Effect on CSCs	Effect on other actors of the NOTCH pathway	Effect on treatment response	Effect on pluripotency
JAG1/NOTCH1	Expression of CD133, CD44, ALDHA1, smad-3 (Fender et al. (2015) c-Myc, Gli1 Singh et al. (2022) and EPCAM Lu et al. (2013)	Upregulation of JAG1/Activation of NOTCH3 by JAG1 Fender et al. (2015)	Higher expression of *HES1*, associated with increased recurrence and poor prognosis after treatment with 5-FU Sun et al. (2017)	Maintenance of pluripotency and heterogeneity in colorectal CSCs through promotion of asymmetric BMI1/LGR5 daughter cell fates Srinivasan et al. (2016)
Soluble JAG1/NOTCH1	Upregulation of MRP-1 and BCL-2 antiapoptotic proteins Fender et al. (2015), Singh et al. (2022)		Enhanced 5-FU resistance through MRP-1 and BCL-2 antiapoptotic proteins Liu et al. (2016)	Increase of sphere forming abilities Zhao et al. (2020b)
Soluble JAG2/NOTCH1 (HES1/HES5)	Modulation of differentiation though CD44, KLR5, SOX9 and NOX1 Rodilla et al. (2009)		Enhanced resistance to Oxaliplatin and SN38 Meng et al. (2009)	Differentiation blockade Rodilla et al. (2009)
DLL1/NOTCH1 (HES1)	Overexpression of drug efflux transporters ABCC1, ABCC2 and P-gp1 Xie et al. (2020)		ABCG2-mediated drug resistance to 5-FU Xie et al. (2020)	
JAG1/NOTCH3 (HES1)	Expression of CD44, OCT4, LGR5 Ying et al. (2015)	Interference with NOTCH1-mediated activation of HES1 through competition for RBP-Jk Beatus et al. (1999)	Enhanced 5-FU resistance Ying et al. (2015)	Increased sphere-forming abilities Ying et al. (2015)
DLL4/NOTCH3	Overexpression of *MUSASHI1* and *ALDHA1* Okano et al. (2005), Pastò et al. (2014)	Upregulation of *NOTCH1* through NUMB inhibition Pastò et al. (2014), Choi et al. (2021)		Enhanced clonogenicity and tumorigenicity in colorectal CSCs Serafin et al. (2011)
(HES1/possibly HEY1 and HEY2 Serafin et al. (2011))		Reduction of NOTCH1 activation by DLL4 Serafin et al. (2011)		Maintenance of pluripotency Ying et al. (2015)
/NOTCH2	ABC transporters Jin et al. (2018), Wang et al. (2019)		-	Promotes sphere forming and metastases inducing abilities in colorectal cancer cells Huang et al. (2017), Jin et al. (2018), Wang et al. (2019)
	Expression of CD44, SOX2, CD133, CD26 Apostol et al. (2013), Huang et al. (2017), Jin et al. (2018), Wang et al. (2019)			Differentiation blockade Rodilla et al. (2009)
	Modulation of differentiation though CD44, KLR5, SOX9 and NOX1 (Rodilla et al., 2009)			
DLL4/NOTCH1		Upregulation of NOTCH3 receptor transcription and activation Serafin et al. (2011), Pastò et al. (2014)	-	
DLL4/NOTCH (HES1)	*ATOH1* and *CHGA* Upregulation Fischer et al. (2011)		Increased tumorigenicity and of recurrence after irinotecan treatment Hoey et al. (2009)	Promotion of self-renewal and sphere forming abilities Fischer et al. (2011)

The global activation of Notch pathway modulates stemness of colorectal CSCs through the repression of differentiation-related genes such as *MUC2* and *ATOH1* along with the inhibition of apoptosis triggered by cell-cycle arrest proteins like p27, fostering the maintenance of self-renewal. However, the effects of Notch signaling in colorectal CSCs go well beyond inhibition of apoptosis. The asymmetric division of slow-cycling and fast-cycling subpopulations of CSCs is NOTCH1-dependent and helps establish CSC heterogeneity by maintaining both slow-cycling, MYC-independent BMI1+ CSCs and fast-cycling, MYC-dependent LGR5+ CSCs (Srinivasan et al., 2016). The unbalanced distribution of NOTCH1 signaling promotes asymmetric BMI1/LGR5 daughter cell fates, which is dependent on the JAG1 ligand and on HES1 and HES5 transcriptional targets of the Notch pathway (Figure 3). This NOTCH1-dependent asymmetrical division has been shown to be directed by epigenetic events such as miRNA, in particular by miR34a which sequesters NOTCH1 mRNA to generate a bimodal NOTCH1 signal which controls the choice between self-renewal *versus* differentiation (Bu et al., 2013), and by chromatin methylation of upstream Notch activators genes through H3K27me3 enrichment, which forms repressive chromatin domains upon STRAP silencing (Bu et al., 2013; Jin et al., 2017). Remarkably, treatment with the GSI DAPT results in reduced frequency of BMI1+/LGR5+ both *in vitro* and *in vivo*, demonstrating the importance of Notch pathway activation for the maintenance of heterogeneity and pluripotency in colorectal CSCs (Srinivasan et al., 2016).

The main effectors of the Notch pathway involved in CRC cell stemness seem to be the ligand/receptor couple JAG1/NOTCH1. The effect of JAG1 and NOTCH1 on pluripotency in colorectal cancer cells was observed *in vitro* where constitutive activation of Notch in colon tumor cell lines resulted in increased expression of EMT and stemness associated proteins. Transduction of constitutively active NOTCH1 or treatment with recombinant Jagged-1 led to expression of CD44, Slug, Smad-3, and induction of JAG1 expression (Fender et al., 2015) (Figure 3). Interestingly, in this study, although NOTCH1 signaling was shown to activate the expression of CD44, Slug, and Smad-3, this was not via JAG1 but rather the consequence of a signaling cascade of other Notch receptors following the induction of JAG1 expression by NOTCH1. The most likely link between JAG1 and CD44 is the NOTCH3 receptor, since the expression of these three proteins are correlated. Moreover, both overexpression of *NOTCH1* and *NOTCH3* results in induction of CD44 and Slug expression (Figure 3). This complementarity between NOTCH1 and NOTCH3 implies that they may both be needed to mediate stemness in colorectal CSCs with overlapping and distinct roles (Clark et al., 2023). Nevertheless, other studies found a similar effect of the activation of NOTCH1 on self-renewal and stemness markers, but with different markers, including CD44, c-Myc, ALDH1, and Gli1, finding at this occasion a direct link between NOTCH1 and CD44 (Figure 3) (Singh et al., 2022).

JAG1 appears to be the main ligand modulating the Notch-related stemness of colorectal CSCs, for instance in APC-deficient adenomas, *JAG1* deletion in LGR5+ CSCs disturbs stem cell niche formation, suggesting that plasticity of these cells is highly dependent on JAG1 (Nakata et al., 2017). Though, the specific interactions of JAG1 resulting in enhanced stemness in CRC cells are not currently clear and may be influenced by the microenvironment. It was reported that JAG1 expression in the cytoplasm is correlated with the expression of NOTCH3 in tumor cells, suggesting an interaction between these two proteins (Serafin et al., 2011). Considering that the conversion of bone marrow-derived mesenchymal stem cells into CAFs is dictated by direct contact through JAG1/Notch pathway activation (Peng et al., 2014), it is plausible that Notch pathway modulation plays a key role in converting surrounding cells into colorectal CAFs, modelling the CSC niche. Moreover, other cells than CAFs, such as endothelial cells (ECs), colocalize with CRC cells in perivascular regions and positively influence stemness in CSCs through the excretion of a soluble form of C-terminally truncated JAG1, without any direct contact (Lu et al., 2013). This soluble form of JAG1 originates from a full-length protein cleaved by the protease ADAM17 and is secreted by ECs, increasing the tumorigenic potential of neighboring CRC cells, as well as their self-renewal. CRC cells in contact with this secreted JAG1 also exhibit an increase in the expression of CD133 and EPCAM markers along with an enhanced ALDH activity. Interestingly, following the cleavage of both JAG1 and JAG2 by ADAM17 in CSCs the truncated form of these ligands are released from the extracellular membrane where they promote CSC phenotype through NOTCH1 activation (Figure 3) (Wang et al., 2016). The crosstalk between the different effectors of the Notch pathway takes place within the CSC microenvironment and involves different receptors according to the cell receiving the signal.

However, JAG1 is not the only ligand of the Notch pathway enhancing the stemness phenotype. For instance, DLL4 was shown to enhance stemness through the activation of the NOTCH3 receptor, resulting in the overexpression of the stemness marker MUSASHI-1 (Okano et al., 2005; Bley et al., 2021) and inhibition of the NUMB protein (Pastò et al., 2014; Choi et al., 2021) in both colorectal cancer cell lines and primary cultures of colorectal cancer metastases (Figure 3). Inhibition of NOTCH3 in these cells via a neutralizing antibody or shRNA reduces sphere-forming abilities, whereas DLL4 stimulation enhances stemness features like ALDHA1 expression, demonstrating an effect of NOTCH3 activation on stemness and self-renewal in CRC cells. In addition to JAG1 and DLL4, the JAG2/NOTCH pathway also exerts a cancer-promoting effect in colorectal cancer cells, as *JAG2* knockdown in colorectal cancer cells inhibits their expression of CD133, decreasing in a similar manner their ability to form spheroids and to induce metastasis (Table 1) (Huang et al., 2017).

Indeed, NOTCH3 was shown to be overexpressed in spheroids derived from colorectal cancer cell lines (WiDr) compared to the parental cell line, confirming the implication of the NOTCH3 receptor in the maintenance of pluripotency in colorectal CSCs (Ying et al., 2015). These results are corroborated by the fact that *NOTCH3* silencing in CRC cells decreases clonogenic capacity *in vitro* and impairs tumorigenicity *in vivo* (Serafin et al., 2011). Interestingly, HES1 is not the only Notch effector suspected of being responsible for this effect since NOTCH3-induced activation or DLL4 stimulation results in a higher expression of HEY1 and HEY2 (Figure 3).

The HES genes cooperatively regulate maintenance and survival of intestinal stem and progenitor cells (Wu et al., 2017; Dzobo et al., 2020). In particular HES1, hypomethylation-linked activation of which leads to tumor cell proliferation and inhibition of differentiation of these tumor cells into intestinal epithelial cells (Kayahara et al., 2003). An *in vivo* study using a Rosa-NOTCH/Cre + mice (Artavanis-Tsakonas et al., 1999; El Marjou et al., 2004; Fre et al., 2005) demonstrated that activation of the NOTCH1 pathway limited the differentiation of intestinal progenitor cells through the activation of HES1 and repression of the mouse atonal homologue Math-1 and neurogenin-3, two proteins coded by essential genes for secretory and enteroendocrine cell lineage specification (Lee et al., 2001; Zine and de Ribaupierre, 2002). In contrast, HEYL negatively regulates metastasis of colorectal tumors in an *in vivo* model seemingly by inhibiting intravasation of metastasis-initiating cells. Interestingly though, HEYL overexpression results in a slight increase in liver metastases after intrasplenic xenotransplantation raising the possibility that HEYL is beneficial for stemness characteristics but detrimental for dissemination of metastatic cells (Weber et al., 2019).

Since Notch pathway mediates pluripotency and proliferation of CSCs, the targeting of Notch signaling might constitute an effective anti-cancer therapy. However, stem and progenitor cells in non-pathological intestinal regions, as well as in tumor cells, undergo differentiation into goblet cells following inhibition of NOTCH1/HES1 signaling, a non-specific targeting of these pathways could cause severe side effects (Fre et al., 2005). Therefore, a strategy to modulate Notch signaling specifically in cancerous cells without affecting healthy cells is needed, which undoubtedly relies on a better comprehension of the fine tuning of Notch pathway.

In conclusion, Notch signaling is a key regulator of stemness and self-renewal in colorectal CSCs. Activation of Notch receptors, notably NOTCH1 and NOTCH3 by their ligands JAG1 and DLL4 results in the repression of differentiation-inducing genes such as *MUC2* or *ATOH1* and inhibition of apoptosis triggered by cell cycle arrest. Members of the HES family, in particular HES1 and HES5, are the main effectors of Notch receptors activation on colorectal CSCs self-renewal, though the HEY effector family is suspected of being partly involved in this phenomenon. Moreover, Notch pathway plays a major role in asymmetrical division of colorectal CSCs and in the maintenance of several pools of pluripotent stem cell. The key implication of Notch signaling in the regulation and maintenance of CRC raises the possibility of its involvement in chemoresistance linked to CSCs and their heterogeneity.

## 8 Notch ligand-receptor specificity and colorectal cancer stem cells

Many studies have described the role of different Notch actors in the regulation of different stem cell properties. We may therefore wonder if Notch ligand-receptor specificity will affect specific stem cell subpopulations or regulate CSC heterogeneity. Indeed, the heterogeneity in Notch receptors, effectors, and pathways, could shape CSC heterogeneity.

Notch pathway is known to modulate stemness and the tumor initiating phenotype in CRC, though results are variable depending on the study, the model and on the actors of Notch pathway observed. Notch signaling is often considered to be relatively simple since only a few proteins are involved. However, diverse signals output originates from the combinations of interactions between the different ligands and receptors. The role of Notch pathway in tumors is highly dependent on the spatial and temporal context of Notch activation, as well as on the status of other signaling pathways in the cells. This contextuality is probably the main reason for which studies observing the impact of different receptors on CRC stemness find a global augmentation of the CSC phenotype despite a non-redundancy or even antagonist effect of the different molecular actors involved.

For instance, a mutually exclusive relationship between NOTCH1 and NOTCH2 protein expression was observed in a CRC cohort of 1,003 patients. In this cohort, data supported the antagonistic roles of NOTCH1 and NOTCH2 in phenotypes of colorectal cancer cells, where NOTCH1 acted as an oncogenic enhancer whereas NOTCH2 played a tumor suppressor role (Chu et al., 2011). Despite this apparent antagonistic functions in primary colorectal cancer (Chu et al., 2011), in non-pathological intestinal epithelium, NOTCH1 and NOTCH2 receptors were shown to operate in a redundant manner for the maintenance of pluripotent progenitors in endothelial crypts (Riccio et al., 2008). Indeed, expression of both of these receptors leads to an increase in the transcriptional factor HES1, participating in cell cycling though transcriptional modulation of CDK inhibitors p27^Kip1^ and p57^Kip2^. This analogous effect of NOTCH1 and -2 has also been found in tumors from Familial Adenomatous Polyposis (FAP) patients (Rodilla et al., 2009) and might be representative of the behavior of these receptors in the conventional adenoma-carcinoma pathway according to several *in vitro* studies (Guilmeau et al., 2010). In this type of colorectal tumors both receptors are activated by JAG1 and participate in blocking differentiation though HES1 but also through several other regulators such as CD44, KLR5, SOX9 and NOX1 (Rodilla et al., 2009).

Studies considering a more exhaustive observation of Notch pathway demonstrated differential expression and specific effects on CSC populations for each receptor. For instance a study revealed that NOTCH1 expression enhanced SOX2 and OCT4 expression, while reducing levels of CD44, whereas NOTCH2 positively influenced all of those factors as well as CD26 (Apostol et al., 2013). NOTCH3 and NOTCH4, may thus decrease CD44 expression, as seen by the upregulation of this marker upon *NOTCH3* and *NOTCH4* knockdown, but have a positive impact on expression of other transcription factors linked to stemness such as cMET, Setmar and CD26 in CRCs. NOTCH2 seems to be the receptor the most expressed in CSCs, followed by NOTCH1, NOTCH3 and lastly, NOTCH4. NOTCH2 expression positively regulates the transcription factors SOX2, OCT4, CD26, CD44 and c-MET, whereas the other receptors have mixed effects on the expression of these factors. Moreover, the link between actors of the Notch pathway can be different according to the presence and absence of partner proteins. For instance, it has been shown that the reduction of NOTCH1 levels inhibited the upregulation of the *NOTCH3* transcript by DLL4 (Serafin et al., 2011). This might be the effect of a direct regulation of NOTCH3 by NOTCH1 activation, as previously shown in leukemia (Palomero et al., 2006; Chadwick et al., 2009) or in some colorectal cancer models (Pastò et al., 2014). However, NOTCH3 ICD might interfere with NOTCH1-mediated activation of HES1 by competing with the NOTCH1 ICD to access the RBP-Jk transcriptional factor modulated by the Notch pathway (Beatus et al., 1999), and by competing for a common activator, DLL4 (Serafin et al., 2011) (Figure 3). This inhibition of the HES1 activation by NOTCH3 might be circumstantial though, since several *in vitro* and *in vivo* studies demonstrated the activation of HES1 by NOTCH3 (Bellavia et al., 2000; Shimizu et al., 2002; Ye et al., 2022).

In colorectal cancer cell lines, as well as in primary cultures of colorectal metastases, a positive link between the activation of NOTCH3 and NOTCH1 receptor has been reported. Indeed, NOTCH3 activation via its binding to the DLL4 ligand triggers inhibition of the NUMB protein, known to inhibit NOTCH-dependent gene expression (Choi et al., 2021). Inhibition of the NUMB protein, in turn, enhances the activation of the NOTCH1 receptor which, via a positive feedback loop, increases NOTCH3 expression and activation (Pastò et al., 2014) (Figure 3).

Notch ligands, particularly JAG1 and DLL4, appear to both enhance stemness-related characteristics. Though, many studies on Notch pathway demonstrated in other pathophysiological contexts distinct or even opposite functions of the JAG1 and DLL4 ligands, especially in the modulation of angiogenesis. Indeed JAG1 and DLL4 ligands are both expressed in the embryonic aorta and both can activate the NOTCH1 receptor but leading respectively to the establishment of the hematopoietic and endothelial cell fates (Benedito et al., 2009; Gama-Norton et al., 2015). This ligand-specific lineage differentiation reinforces the existence of a ligand-receptor specificity in Notch signaling. Notch signaling specificity is impacted by Notch ligands crosstalk. An example of such a crosstalk between Notch ligands occurs in the intestinal epithelium, where inhibition of DLL4 or DLL1 does not impact intestinal stem cells, albeit their simultaneous inactivation inhibits pluripotency and results in loss of stem cells (Olfm4+, Lgr5+, and Ascl2+) (Pellegrinet et al., 2011).

HES1 is dominant during embryonic and neonatal stages, whereas the genes of HES-1, -3 and -5 cooperate to regulate adult intestinal homeostasis (Ueo et al., 2012). Though, the presence of LGR5^+^ cells in the intestinal tract of Hes-1, -3 and -5 cKO mice comparable to WT mice indicate that the HES transcription factors are not the only targets of Notch signaling involved in pluripotency maintenance.

Despite the numerous studies demonstrating the different effect of Notch pathway on colorectal CSCs, the crosstalk between the different actors of the Notch pathway and the specific effects they have render studies on the impact of these individual proteins on stemness inapplicable. Few studies focus on several Notch pathway proteins, which is needed to provide a more accurate insight into the impact of Notch pathway on stemness and self-renewal. These limitations and several other parameters need to be considered when exploring the relationship between Notch pathway, stemness phenotypes, CSCs heterogeneity and resistance to treatments.

**FIGURE 4:**
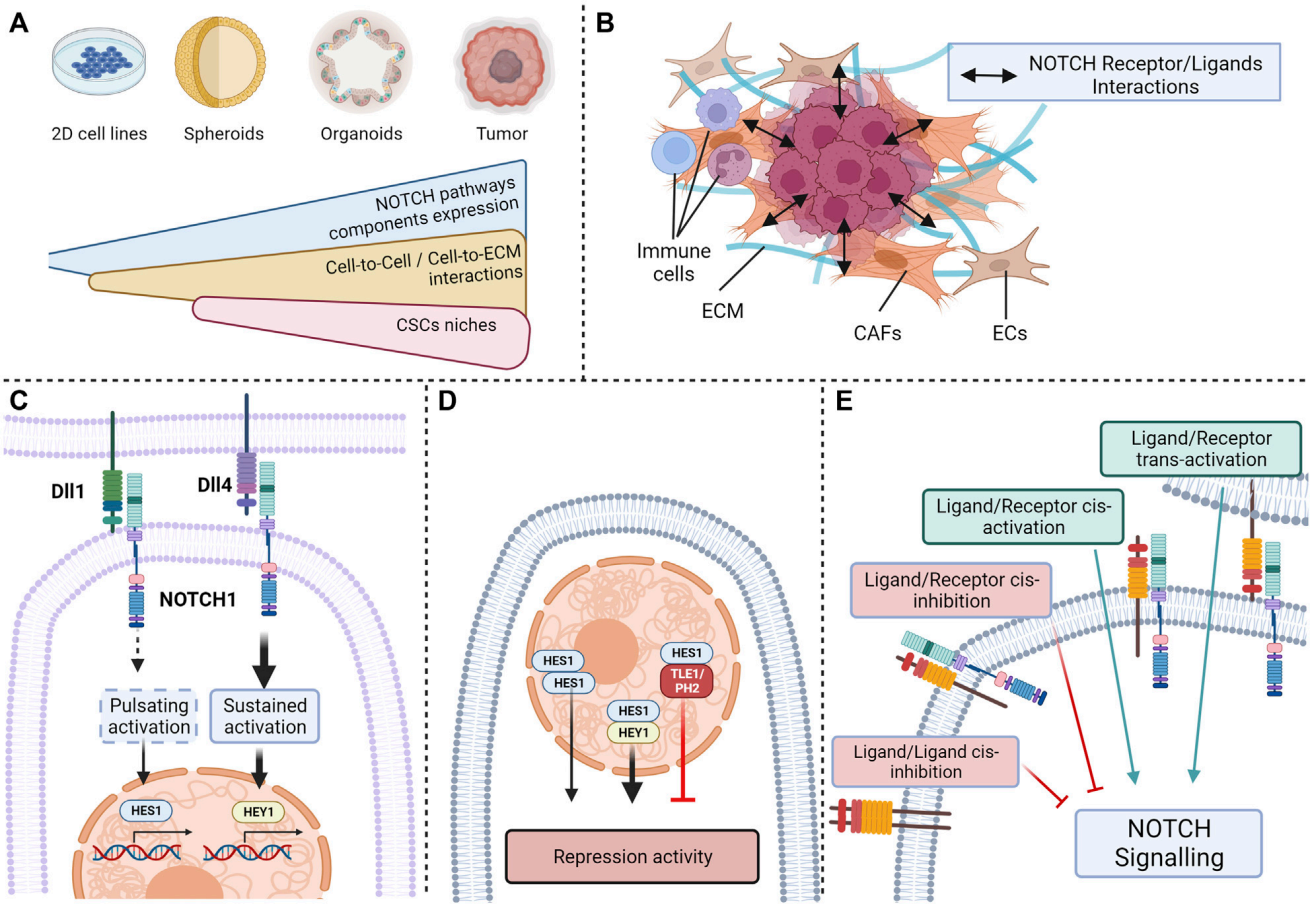
Current limitations in the study of NOTCH ligand/receptor complexity: **(A)** Variable expression of NOTCH pathway components depending on the complexity of the model; **(B)** Interactions between NOTCH pathway actors and extracellular-matrix or neighboring cells; **(C)** Activation of the same receptors by different ligands resulting in different outcomes; **(D)** NOTCH effector activation resulting in different outcomes depending on the presence or absence of nuclear partners; **(E)** Cis- and trans-activation and -inhibition of NOTCH receptors (Created with BioRender.com).

One of the main limits encountered in studies investigating Notch pathway is the low endogenous activity of Notch signaling in many commonly used CRC cell lines in the absence of cytotoxic chemotherapy (Akiyoshi et al., 2008; Zhang et al., 2008; Meng et al., 2009). Although Notch signaling seems decisive in adenoma formation and CRC tumorigenesis, this low level of expression does not accurately reflect the interactions and implications of the actors of Notch pathway in tumors (Marconi et al., 2021). Consequently, the impact of Notch pathway modulation was shown to be stronger in three-dimensional models like colorectal spheroids than in parental cell lines (Huang et al., 2015a). Notch signaling being dependent on cell-to-cell interactions or cell-to-ECM interactions, the use of immortalized two-dimensional cell lines is not optimal to observe the complex dynamics of Notch signaling proteins. For instance, a link between Notch signaling, more precisely on HEY2 expression and the tenascin-C, an hexameric glycoprotein from the ECM binding to fibronectin, periostin and integrins, was described (Oskarsson et al., 2011). Indeed, tenascin-C protects *HEY2* transcriptional expression from inhibition STAT5, enhancing stemness phenotype and pluripotency of metastatic breast cancer cells. This positive effect of the ECM protein tenascin-c on Notch signaling and stemness has also been reported in glioma, this time on expression of JAG1, ADAMTS15, and activation of NOTCH1 and NOTCH2 receptors (Sarkar et al., 2017). Such a relationship between ECM components, Notch pathway and stemness necessitates models able to reproduce these interactions, which is not the case of most 2D models. This statement about the use of two-dimensional cell lines is relevant for the studies of Notch signaling on stemness and self-renewal, given the major implications of the microenvironment and of the interactions between cells in these features (Le et al., 2008; Farahani et al., 2014; Avnet and Cortini, 2016; AlMusawi et al., 2021). A solution explored by several teams is the preferential use of three-dimensional models such as organoids or spheroids that are more suitable for studying complex interactions (Huang et al., 2015a; Högström et al., 2018; Jackstadt et al., 2019). For instance, organoids generated from induced pluripotent stem cells can reproduce the transient expression patterns of HES1, reported in embryonic stem cells and deemed critical for physiological Notch signaling (Shimojo et al., 2008; Trujillo et al., 2019; Marconi et al., 2021). Such models are more amenable to reproducing the complex interactions between the molecular actors of these pathways occurring within the cell and with its microenvironment (Figure 4A).

Another parameter from the cell microenvironment that could explain the ligand/receptor specificity observed in Notch pathway is the crosstalk between tumor cells and surrounding cells such as endothelial cells, CAFs or immune cells (Figure 4B). Such an example was given above with the effect of soluble JAG1 secreted from endothelial cells on CSCs (Lu et al., 2013). These interactions between CAFs and CRC cells could partially explain differences in Notch activities observed according to the localization of tumor cells. Indeed, in a similar manner to pluripotent cells, disparities of Notch activation have been observed between cells populating the edge of the tumor, with low signs of NOTCH activation, and cells originating from the center, with a high expression of NOTCH1 ICD (Schmidt et al., 2018). Regulation of Notch signaling by the tumor architecture, in this case the activation of *HEYL* transcription by JAG1 and JAG2 expression in neighboring cells, is also observed in other types of cancers, such as basal cell carcinoma (Eberl et al., 2018) and might be an important criterion in CRC. In addition, immune cells may drive Notch signaling at the edge of the tumor, as for breast cancers, where the stimulation of JAG1/NOTCH interactions by cytokine like IL-6 triggers a partial mesenchymal phenotype and an increase in stem-like characteristics (Bocci et al., 2019). Another example of this crosstalk between CSCs and immune cells is the effect of mammary stem cell-DLL1 on adjacent macrophages observed in the mammary gland, where NOTCH1,-3 and -4 activation in macrophages is triggered by this interaction results in the production of Wnt ligands stimulating pluripotency in mammary stem cells (Chakrabarti et al., 2018).

Furthermore, it was recently described that the different ligands of the Notch pathway can mediate distinct targets through their interactions with a common Notch receptor. For instance, Nandagopal et al. demonstrated by quantitative single cell imaging that DLL1 and DLL4 ligands were have different effects on cell fate through their activation of the NOTCH1 receptor (Nandagopal et al., 2018). DLL1 triggered a pulsating activation of the NOTCH1 receptor, resulting in the activation of the HES1 transcriptional target, whereas Dll4 led to a sustained activation of the receptor causing HEY1 activation, these targets inducing specific cell fates. The dynamics of NICD production is a decisive parameter for signals triggered by a Notch receptor, but the intensity of the signal has also been shown to influence downstream pathways and to be dependent on the ligand (Figure 4C). A good illustration of this factor is the difference in the strength of signal triggered by Jag1 and Dll4 on hematopoietic stem cells (HSC) showed by Gama-Norton et al. with Notch1 activation trap mouse models with different sensitivity (Gama-Norton et al., 2015). Jag1 stimulates a low activation of Notch1 receptor, whereas Dll4 triggers a stronger Notch1 activation. This antagonistic relationship between jagged and delta ligand receptors based on the strength of Notch signaling has been observed in several physiological features (Benedito et al., 2009; Petrovic et al., 2015) and may be implicated in stemness.

The patterns of expression of effector proteins HEY and HES also seem to be crucial in outcomes triggered by Notch receptors. HES and HEY can be individually expressed in some cells, but they can also be co-expressed upon Notch receptors activation. HES and HEY homodimers and heterodimers bind similar DNA sequences, but with different repression activities levels (Leimeister et al., 2000; Iso et al., 2001; Van Wayenbergh et al., 2003; Kageyama et al., 2007). In addition, other transcriptional regulators such as TLE1 or PHB2 can bind to HES1, thus repressing complexes (Rohena-Rivera et al., 2018). The complexity of these effectors results in different phenotypical outcomes triggered by Notch pathway (Figure 4D).

Finally, the plurality of interactions between the receptors and ligands if a confusion factor that may impact Notch pathway effect on stemness-related signaling. Indeed, different interactions between Notch receptors are possible depending on the cell expressing the ligand. Ligand/receptor trans-interaction, *i.e.,* between neighboring cells, results in classical trans-activation, whereas ligand/receptor cis-interaction, *i.e.,* within a cell, can also occur (LeBon et al., 2014). These particular types of interactions can either result in Notch receptor cis-activation (Formosa-Jordan and Ibañes, 2014; Nandagopal et al., 2019) or Notch cis-inhibition (del Álamo et al., 2011; Miller et al., 2009). Cis-activation has been observed through single molecule HCR-FISH detection of Notch targets (Nandagopal et al., 2019), whereas cis-inhibition, showed by Miller et al. using genetically modified *drosophila* models, decreases the capacity of a cell to receive signals from neighboring cells (Meir et al., 2002). In contrast, cis-activation affects the survival of neural stem cells, suggesting that self-renewing cells can sustain themselves, activating their own Notch signaling, in particular NOTCH1 and NOTCH2, in an autocrine manner (Nandagopal et al., 2019). In addition to these ligand/receptor interactions, ligand/ligand interactions have been observed through mutational analysis by Chen D et al., and highlight a type of cis-inhibition which is triggered by ligand dimers (Chen et al., 2023). Together, these finely balanced mechanisms dictate the strength, the flow, and the specificity of Notch signaling. This multitude of potential interactions complexifies the link between the expression of Notch receptors and ligands, and the resulting signaling on CSCs and has to be considered (Figure 4E).

Signaling resulting from the Notch pathway is a complex network of different receptors and ligands, the interactions of which coordinate distinct outcomes though activation of various effectors, underlying ligand and receptor specificity of the Notch pathway in colorectal CSCs. This complexity relies on interaction between CSCs, the microenvironment and neighboring cells, as well as the numerous types of interactions between the different actors of Notch pathway. This complex intricacy might be the reason, at least partially, contradictory results obtained by studies attempting to decipher the role of Notch pathway in colorectal CSCs. Though, another explanation is plausible, indeed, the multiple effects observed of the different Notch pathway actors on CSCs might be the reflection of Notch signaling impact on CSCs heterogeneity. As previously mentioned, CSC clusters exhibiting various phenotypic traits co-exist in CRC tumors, the regulation and tuning of these different clusters may be directly regulated through the ligand/receptor specificity of pathways such as Notch, as suggested by the asymmetrical division of slow- and fast-cycling CSCs directed by distribution of NOTCH1. Approaches such as genome-scale studies (Guruharsha et al., 2012), spatial transcriptomics (Cang et al., 2023), and computational modeling such as agent-based model (Reynolds et al., 2019) could be used to better determine the complexity of Notch pathway in adequation with CSCs heterogeneity.

## 9 Notch pathway ligand-receptor specificity and colorectal CSC: implications for post-treatment recurrence and chemoresistance

Similarities have been found between colorectal CSC-enriched cell lines via 3D-colonosphere culture and chemoresistant CRCs. Despite differences in these populations, these techniques have both been shown to enhance the proportion of CSCs (Vinogradov and Wei, 2012). Colon cancer spheroids and chemoresistant CRC cells both express the stemness markers, CD133 and CD44, and exhibited equivalent phenotypes. Notch signaling, notably the NOTCH1 pathway, was reported to play a major role in colorectal spheroids and in chemoresistant cell lines, suggesting that Notch activation is involved in maintaining a phenotypic characteristic common to spheroids and chemoresistant cells (Huang et al., 2015b). As presented above, cells able to self-renew such as CSCs, TICs and DTP exhibit an increased resistance to treatment and numerous studies present a direct link between Notch-dependent stemness in these cells and their resistance to treatments. The main reported associations between specific Notch ligand-receptor pairs, self-renewal and treatment response are summarized in Table 1.

For instance, HES1, often considered as the best marker of Notch pathway activation, is highly expressed in stage II CRC patients with higher recurrence rate and poor prognosis after treatment with 5-FU. These effects were reproduced *in vitro,* where cell lines overexpressing HES1 display higher 5-FU resistance (Sun et al., 2017). HES1 expression and the resulting chemoresistance is correlated with EMT signaling and increased expression of drug efflux transporters like ABCC1, ABCC2 and P-gp1 (Figure 3). This effect of HES1 on ABC transporters might be modulated by the DLL1-activation of NOTCH1 receptor. Indeed, the DLL1/NOTCH1 couple modulate the ABCG2-mediated drug resistance to 5-FU in colon cancer side population cells, which are a small subpopulation of cells exhibiting stemness characteristics and enhanced drug resistance (Figure 3) (Xie et al., 2020).

Indeed, NOTCH1 activity was shown to intensify resistance of CRC cells to 5-FU through the upregulation of MRP-1 and BCL-2 antiapoptotic proteins (Liu et al., 2016) (Figure 3). Moreover, the NOTCH1 pathway is linked to resistance to other drugs, as its activation, presumably by JAG1, is associated with resistance to Methotrexate, an inhibitor of dihydrofolate reductase used as a chemotherapeutic agent in colorectal cancer. This resistance is associated with stem-cell like characteristics such as an increased expression of markers CD166, CD26, CD44 and CXCR4, as well as enhanced sphere forming abilities (Zhao Q. et al., 2020). NOTCH1 activation is involved in resistance to other therapeutic agents such as oxaliplatin, 5-fluorouracil (5-FU), or SN-38 (the active agent of irinotecan treatment) (Meng et al., 2009). Indeed, these therapies all lead to an over-activation of NOTCH1 characterized by an increase in NICD and HES1 protein levels due to an increase in gamma-secretase activity.

Several studies reported a negative correlation between *NOTCH2* expression in tumor and tumor stage, as well differentiated carcinomas were shown to harbor a higher *NOTCH2* expression compared to poorly differientiated ones (Chu et al., 2009), suggesting than NOTCH2 may be a marker of differentiation rather than of stemness. However, NOTCH2 signaling has been shown to induce the expression of stemness-related genes such as *CD133* and *SOX2*, to promote resistance to chemotherapies through the expression of drug transporters like ATP-binding cassette and enhance sphere forming abilities of CRC cells (Jin et al., 2018; Wang et al., 2019) (Figure 3). The implication of NOTCH2 in chemoresistance and pluripotency of CRC cells remains ambiguous and necessitates further research.

In the case of NOTCH3, its suppression *in vitro* leads to a decrease in sphere-forming abilities, in *OCT4* and *LGR5* expression, and an increased sensitivity to 5-FU treatment (Ying et al., 2015) (Figure 3). Interestingly, in this model, the inhibition of NOTCH1 had no effect on stemness characteristics, such as sphere forming properties, expression of stemness markers or resistance to treatment, suggesting that the implication of Notch receptors and actors of the Notch pathway in stemness-linked chemoresistance is highly contextual.

Although the precise implications of each protein of Notch signaling in chemoresistance remains elusive, evidence indicates that Notch pathway activation results in enhanced chemoresistance in CRC (Vinson et al., 2016). The use of GSI has a synergistic effect with most therapeutic agents used for the treatment of CRC including oxaliplatin, SN-38, and 5-FU (Meng et al., 2009), demonstrating that the overall activation of Notch pathway results in resistance to common chemotherapies used in CRC. In the specific context of stemness-linked chemoresistance, treatment of colorectal CSCs with GSI in combination with irinotecan results in a decrease in stemness marker, like ALDH, and a significant inhibition of tumor regrowth following treatment in a fraction of CRC tumors exhibiting high activation of Notch pathway (Arcaroli et al., 2012).

The use of GSI also enhanced mitotic arrest and apoptosis of colon cancer cells following taxane treatments like paclitaxel (Akiyoshi et al., 2008). However, this effect of gamma-secretase is believed to be independent of the Notch pathway since silencing of genes coding for NOTCH1, -2 and -3 receptors does not improve paclitaxel efficacy.

As mentioned above, DLL4 has a positive impact on stemness-related characteristic and its inhibition, for instance via a neutralizing antibody, results in the reduction of self-renewal and of tumor growth *in vivo* (Fischer et al., 2011). However, when combined with irinotecan, these effects are exacerbated and anti-DLL4 enhances cell death triggered by irinotecan by reducing levels of anti-apoptotic genes, such as HSPA6, and enhancing expression of proapoptotic genes like *PDCD4*. Blockade of DLL4 was reported to result *in vivo* in a decrease in tumorigenicity and of recurrence after treatment with irinotecan (Hoey et al., 2009).

Targeting the Notch pathway has emerged as a promising strategy for addressing two critical aspects of colorectal cancer treatment: chemoresistance and tumor recurrence. Chemoresistance poses a significant challenge in cancer therapy, leading to treatment failure and recurrence. In this context, the Notch pathway plays a pivotal role, particularly in colorectal cancer stem cells (CSCs), which contribute to treatment resistance and tumor relapse.

Studies have revealed that Notch signaling promotes the survival and maintenance of chemoresistant CSCs. It has been shown for instance that an elevated *JAG1* expression was associated with poorer prognosis and chemoresistance in CRC patients (Kim et al., 2019). By inhibiting the Notch pathway, it is possible to restore sensitivity to chemotherapy agents, thereby overcoming chemoresistance and improving treatment outcomes. Targeting Notch disrupts CSC self-renewal, enhances drug-induced cell death, and sensitizes CSCs to the cytotoxic effects of chemotherapy drugs (Takebe et al., 2015; Morel et al., 2017).

Preclinical studies have explored the combination of Notch pathway inhibitors with conventional chemotherapy agents to overcome chemoresistance and prevent recurrence. For instance, combining gamma-secretase inhibitors (GSIs) with chemotherapeutic agents, such as 5-fluorouracil (5-FU), has demonstrated synergistic effects in reducing tumor growth and overcoming resistance in colorectal cancer models (Meng et al., 2009). Inhibition of Notch signaling sensitizes CSCs to the cytotoxic effects of 5-FU, leading to enhanced cell death and improved treatment response. Simultaneously, it hampers the regrowth and self-renewal potential of residual cancer cells, minimizing the risk of recurrence (Strosberg et al., 2012).

Clinical trials investigating the combination of Notch pathway inhibitors with chemotherapy in chemoresistant colorectal cancer and its impact on recurrence prevention are underway (Takebe et al., 2014). Targeting the Notch pathway not only addresses chemoresistance but also holds potential in reducing the likelihood of tumor recurrence, offering a comprehensive therapeutic strategy for colorectal cancer patients (Suman et al., 2014).

In summary, Notch signaling generally leads to a higher expression of detoxifying enzymes such as ALDH, drug efflux transporters like ABCC1 and ABCC2, as well as an upregulation of anti-apoptotic genes and proteins like *PDCD4*, *HSPA6*, BCL-2 and MRP-1. The protection of self-renewing cells following Notch pathway activation upon treatment leads to CRC recurrence and may be targeted to limit this event. The main contributors to this phenomenon are NOTCH1 and NOTCH3 receptors, the role of NOTCH2 being incompletely understood. Most of the Notch ligands studied seem to enhance chemoresistance, DLL1, DLL4 and JAG1 being the principal activators in this event. Moreover, the asymmetrical division of CSC subtypes orchestrated by Notch pathway contributes to the constitution of a slow-cycling CSC population, contributing to a pool of resistant stem cells.

Though, the studies exploring the implications of Notch pathway on chemoresistance and recurrence suffer from the same disparities as studies on CSCs. Indeed, as we have seen, Notch pathway components all have different effects on chemoresistance and CSCs related recurrence. This might be an indication of the regulation of CSCs different clusters through the ligand/receptor specificity of Notch pathway.

## 10 Conclusion

CSCs have been extensively studied in the context of chemoresistance and recurrence after treatment, these cells being considered as the main reasons for tumoral heterogeneity, and the ensuing resistance and recurrence. Indeed, these subpopulations exhibit features allowing multi drug resistance such as their proliferative quiescence, the high expression of DNA damage repair proteins, of detoxifying enzymes and of ABC transporters.

NOTCH pathway activation seems to be a key regulator of stemness as well as chemoresistance in CRC. Despite the lack of precise mechanisms linking NOTCH pathway activation to these features, an increasing amount of evidence supports the implication of NOTCH pathway proteins in stemness and chemoresistance via the modulation of asymmetric division, cell-cycle, and expression of anti-apoptotic proteins, ABC transporters and detoxifying enzyme. In addition to being an architect of intra-tumoral heterogeneity through its control of CSC asymmetrical division, the NOTCH pathway is a direct regulator of CSCs-related chemoresistance.

Though, considering the heterogeneity of CRC cells able to self-renew and the numerous molecular actors modulating NOTCH pathway, several parameters need to be taken into consideration to gain further insight into the implication of NOTCH pathway in these events and obtain reliable studies. Among these parameters, the microenvironment, and the general context in which NOTCH pathway proteins act (expression, interactions partners, and localization) are critical. A better characterization of these pathways, of interactions between these molecules, and of their implications in colorectal CSCs would allow the development of drugs targeting specific branches of the NOTCH pathway to be used in combination with commonly used chemotherapy or targeted therapies to prevent chemoresistance and recurrence in colorectal cancer.

Thus, to reconcile conflicting results between studies addressing the impact of NOTCH on stemness and recurrence in CRC, we argue that additional work should take into consideration NOTCH ligand/receptor specificity and its complex impact on CSC heterogeneity.
